# 针对微量血浆样本的N-糖肽规模化的富集鉴定方法

**DOI:** 10.3724/SP.J.1123.2025.04004

**Published:** 2025-09-08

**Authors:** Xinyi YANG, Weijie QIN

**Affiliations:** 1.安徽医科大学基础医学院，安徽 合肥 230032; 1. School of Basic Medical Science，Anhui Medical University，Hefei 230032，China; 2.医学蛋白质组全国重点实验室，国家蛋白质科学中心 （北京），北京蛋白质组研究中心，军事科学院军事医学研究院，北京 102206; 2. State Key Laboratory of Medical Proteomics，National Center for Protein Sciences （Beijing），Beijing Proteome Research Center，Beijing Institute of Lifeomics，Beijing 102206，China

**Keywords:** 富集, 质谱, 蛋白质组, 亲水相互作用色谱法, N-糖肽, 血浆, enrichment, mass spectrometry （MS）, proteome, hydrophilic interaction chromatography （HILIC）, N-glycopeptide, plasma

## Abstract

血浆是血液的重要组成部分，它作为临床蛋白质组学研究的重要样本，蕴含着丰富的生理与病理信息，是发现疾病相关生物标志物的理想来源。蛋白质N-糖基化作为一种关键的翻译后修饰，广泛参与细胞间通讯、免疫调节和信号转导等生物学过程，其异常变化与肿瘤、自身免疫疾病和神经退行性疾病等多种病理状态密切相关。因此，N-糖基化蛋白质组学在生物标志物和药物靶点开发中具有重要价值。然而，由于N-糖肽在生物样本中的丰度较低，且质谱分析时易受高丰度非修饰肽段的信号抑制，因此，在质谱检测前对其进行高效富集是实现深度N-糖蛋白质组覆盖的关键挑战，特别是对于微量血浆样本目前尚缺乏深入研究。本工作针对微量血浆N-糖肽构建了一种高效富集方法和高灵敏度质谱分析策略，首先通过优化亲水相互作用色谱法（HILIC）填料固定相化学组成、孔径大小等关键参数与N-糖肽的洗脱梯度，得到具有高选择性的富集方案，并结合基于平行累积序列碎片技术（PASEF）高分辨质谱仪Tims TOF Pro 2与高质量精度的Orbitrap Lumos的双平台互补分析，显著提升了N-糖肽的鉴定深度，在仅使用20 μg血浆肽段（等效0.5 μL全血浆）的条件下，通过HILIC富集得到了2 962条完整N-糖肽，显著提高了N-糖肽鉴定的灵敏度，成功填补了微量血浆N-糖肽富集技术的空白，也为基于血浆N-糖蛋白质组学的精准医学研究提供了可靠的分析平台，为疾病生物标志物的发现提供了技术支持。

## Abstract (en)

Blood， which forms part of the systemic circulatory system， contains proteins from various tissues and organs. Hence， blood samples are ideal vehicles for studying diseases and physiological states. Plasma is an important component of blood and is essential for clinical proteomics research. Plasma contains rich physiological and pathological information； consequently， it is an ideal medium for discovering disease-related biomarkers. Protein N-glycosylation is a key post-translational modification route. This route is widely involved in biological processes such as intercellular communication， immune regulation， and signal transduction. Changes resulting from aberrant N-glycosylation are closely associated with various pathological conditions， including autoimmune and neurodegenerative diseases and tumors. Hence， N-glycosylation proteomics is highly valuable during biomarker and drug-target development. However， efficiently enriching N-glycopeptides in biological samples before detection by mass spectrometry （MS） is difficult. This is because the highly abundant unmodified peptides result in signal suppression. Consequently， achieving deep N-glycoproteomic coverage is a key challenge， particularly for trace plasma samples， for which in-depth studies are currently lacking. In this study， we developed a strategy for comprehensively profiling trace N-glycopeptides in plasma. This includes an efficient enrichment method in combination with highly sensitive MS. The developed approach integrates glycopeptide enrichment using advanced hydrophilic interaction liquid chromatography （HILIC） with state-of-the-art MS platforms. This significantly enhances detection depth and sensitivity during N-glycosylation analysis using minimal plasma volumes. Selectivity and efficiency during N-glycopeptide enrichment were maximized by systematically optimizing key HILIC-packed stationary-phase parameters. These parameters include chemical composition， pore size， and surface modification. Additionally， the elution gradient was fine-tuned to improve glycopeptide recovery. This optimization process delivered high N-glycopeptide specificity， even in complex plasma matrices. To overcome the limitations of single-platform MS， we implemented a complementary dual-platform strategy. This strategy combines the high-speed， high-resolution capabilities of the Tims TOF Pro 2 instrument with the ultra-high mass accuracy and resolution of the Orbitrap Lumos spectrometer. The former instrument facilitates the rapid and sensitive identification of glycopeptides， particularly for low-abundance species. It exploits the trapped ion mobility spectrometry （TIMS） and parallel accumulated sequential fragmentation （PASEF） technology. The Orbitrap Lumos provides exceptional mass accuracy and high-resolution MS/MS spectra that enable confident glycopeptide structural characterization. This synergistic approach significantly expands the N-glycopeptide identification depth and ensures comprehensive glycosylation-site and glycan-composition coverage. The developed optimized workflow successfully identified 2 962 intact N-glycopeptides using only 20 μg of plasma peptides （equivalent to 0.5 μL of whole plasma）. This set a new benchmark for sensitivity in the micro-volume plasma glycoproteome field. This achievement addresses a critical gap， where conventional methods typically require much larger sample volumes. This limits their applicability to clinical and precision medicine settings where sample availability is restricted. The developed platform provides a robust and reliable analytical framework for plasma N-glycoproteomics with significant implications for precision medicine. This method facilitates large-scale clinical studies by enabling highly sensitive glycopeptide profiling from very small plasma volumes. This included the longitudinal monitoring of disease progression and therapeutic responses. Furthermore， it offers a powerful tool for discovering novel N-glycosylation-based biomarkers for use in early disease diagnosis， prognosis， and personalized treatment strategies. In summary， this study advances the technical capabilities of plasma N-glycoproteomics. Additionally， it facilitates the broader use of plasma N-glycoproteomics in biomedical research and clinical diagnostics.

血浆N-糖蛋白质组研究在生物医学领域具有重要意义，不仅能反映全身生理病理状态，还可用于监测疾病进展和治疗响应。血浆作为人体循环系统的重要组成部分，含有丰富的蛋白质，其中超过50%的蛋白质存在糖基化修饰。N-糖基化作为最常见的蛋白质翻译后修饰之一，在细胞识别、免疫应答、信号传导等生命过程中发挥关键作用^［1-3］^。N-糖型的异常变化与癌症、糖尿病、神经退行性等多种病理状态密切相关^［4-7］^。血浆蛋白的异常糖基化已被证实是多种疾病的早期分子事件^［1，2，5，8-11］^。例如，肝癌患者血清中甲胎蛋白（*α*-fetoprotein）的核心岩藻糖基化水平显著升高^［12，13］^，而阿尔茨海默病患者血浆中载脂蛋白E的唾液酸化修饰则呈现特征性降低^［14］^。然而，血浆N-糖肽的检测面临巨大挑战：一方面，高丰度蛋白（如白蛋白、免疫球蛋白）占血浆总蛋白的90%以上，导致低丰度N-糖肽信号被掩盖^［15］^；另一方面，N-糖肽的离子化效率低且存在微观异质性，质谱检测灵敏度显著受限^［16］^。因此，发展高效的N-糖肽富集方法和高灵敏度质谱鉴定策略对于疾病标志物发现和精准医学研究具有重要价值^［5］^。目前主流的N-糖肽富集策略包括：亲水相互作用色谱法（HILIC）^［17-21］^、硼酸亲和法^［22-24］^、凝集素亲和层析法^［17，25］^和肼化学捕获法。由于临床血浆样本组成高度复杂且通常采集量有限，传统方法如凝集素和硼酸富集法亲和力较弱，富集选择性不佳，且往往需要数百微升血浆起始量；而肼化学捕获易导致N-糖链结构破坏，均难以满足微量血浆样本的完整N-糖肽分析需求^［26，27］^。HILIC富集基于N-糖肽比非糖肽更强的亲水性实现分离，具有选择性高、不破坏N-糖链结构和质谱兼容性好等优势，但也存在需要较大样品起始量才能获得良好富集效果的局限。针对以上问题，本研究通过对N-糖肽富集的肽段起始量、HILIC填料、N-糖肽的洗脱梯度以及检测仪器进行全流程方法优化（图1），以实现微量血浆起始量样本中N-糖肽的高效富集和高灵敏度鉴定，为疾病的糖蛋白标志物筛查提供新工具，助力实现疾病的早期无创诊断和个体化治疗监测。

**图1:**
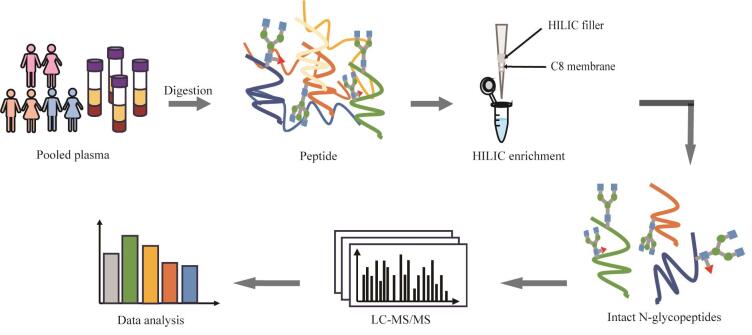
血浆N-糖肽富集实验流程

## 1 实验部分

### 1.1 仪器与试剂

Orbitrap Fusion^TM^ Lumos^TM^ Tribrid^TM^质谱仪、Ultimate3000液相色谱系统、Fresco17高速离心机、NanoDrop^TM ^One微量紫外-可见（UV-Vis）分光光度计、Pierce^TM ^BCA（二喹啉甲酸）试剂盒均购自美国Thermo公司；Bruker Tims TOF Pro 2质谱仪购自美国Bruker公司；CPA225D分析天平购自德国Sartorius公司；磺酸甜菜碱型（sulfobetaine type）孔径12 nm的HILIC填料购自上海菲齐生物科技有限公司；磺酸甜菜碱型孔径20 nm的HILIC填料、过滤器辅助的样品制备（filter-aided sample preparation，FASP）离心管购自德国Merck公司；酰胺型（amide type）HILIC填料购自天津博纳艾杰尔科技有限公司。

氯乙酰胺（CAA）、三（2-羧乙基）膦（TCEP）、四乙基溴化铵（TEAB）、三氟乙酸（TFA）、乙腈（ACN）、尿素（urea，UA）、甲酸（FA）均购自美国Sigma公司；胰蛋白酶（trypsin）购自美国Promega公司；胞内蛋白酶（Lys-C）购自美国PTM公司；三羟甲基氨基甲烷盐酸盐（Tris-HCl）购自北京酷来搏科技有限公司；Empore disk C8固相萃取膜购自美国CDS Analytical公司；Axygen^®^ 20 µL超微量移液器吸头（Tip吸头）购自美国Corning公司。

10人混合血浆样本由10名健康志愿者提供：5位女性和5位男性；单人血浆：从10名健康志愿者血浆样本中选取3名健康女性志愿者和3名健康男性志愿者。健康志愿者混合血浆样本所涉及的志愿者均已签署知情同意书，由北京蛋白质组研究中心伦理委员会审批通过，审批号为20200410-01HL。

### 1.2 溶液配制

8 mol/L UA溶液：移取2.88 g UA、600 μL Tris-HCl至3 mL水中，超声溶解，再加水补充至6 mL，混匀。BCA工作液：将BCA试剂盒中的试剂A与试剂B按照50∶1的体积比混合。血浆BCA法待测样品：取1 μL单人血浆，用水稀释100倍。N-糖肽洗涤/结合缓冲液：将ACN、TFA和水按照体积比80∶1∶19的比例进行混合。N-糖肽洗脱缓冲液：将ACN、TFA和水分别按照体积比65∶1∶34和20∶1∶79的比例混合配制洗脱缓冲液1与2；将TFA和水按照体积比1∶999的比例混合配制洗脱缓冲液3。

### 1.3 血浆蛋白含量BCA法定量

通过BCA法对6例健康单人血浆（3名女性，3名男性）进行含量测定，首先梯度稀释BSA标准品，配制7种不同质量浓度（2 000、1 500、1 000、750、500、250和125 μg/mL）的BSA标准品溶液，在96孔板内每个孔中加入200 μL BCA工作液和10 μL BSA标准品溶液/血浆待测样品，轻摇混匀，在37 ℃孵育30 min，冷却至室温，使用酶标仪在562 nm测定吸光值，通过已知浓度的BSA稀释液的吸光值与蛋白浓度绘制标准曲线，再根据标准曲线定量血浆待测样品的蛋白浓度。最终结果如下：3名女性的血浆样本蛋白含量分别为131.03、143.57和177.50 μg/μL，3名男性的血浆蛋白含量分别为120.70、94.27和113.13 μg/μL。

### 1.4 血浆蛋白FASP酶切

将10人混合血浆样本放置于室温，溶解后取2 μL加入200 μL 8 mol/L UA溶液混合，再将混合溶液转移至30 kD超滤管中，转速14 000 g，离心15 min，加入终浓度10 mmol/L的TCEP与40 mmol/L的CAA（8 mol/L UA配制，现用现配），在室温暗处反应45 min，将其转移至离心机，转速14 000 g，离心15 min，去除还原烷基化试剂，加入200 μL UA清洗一次，放入离心机，转速14 000 g，离心20 min，最后使用50 mmol/L TEAB缓冲液200 μL，转速14 000 g，15 min离心，置换超滤管中残留的UA及其他杂质（重复置换步骤4次，防止残留尿素等氨基物质），最后更换新的收集管收集离心液体。在超滤管中加入溶解有2 μg Lys-C酶的200 μL 100 mmol/L TEAB缓冲液，37 ℃反应4 h，补加2 μg胰酶继续反应过夜，酶切结束，将超滤管放入离心机，转速14 000 g，15 min离心，回收下层液体，超滤管中再加入200 μL水，再超滤一次，合并两次超滤下来的液体，冻干至10~20 μL后使用NanoDrop^TM^ One微量UV-Vis分光光度计测定浓度，按5、10、20、40 μg分装肽段，继续冻干，放置-80 ℃保存待用。

### 1.5 HILIC糖肽富集

首先使用1 mg HILIC材料颗粒和两层C8 Empore膜填充Tip吸头，制备HILIC富集Tip吸头。肽段样品在洗涤/结合缓冲液中重悬，然后装入HILIC富集吸头，3 000 r/min，离心2 min，重复样本装填步骤3次。然后使用50 μL 5×洗涤/结合缓冲液洗涤吸头，以去除非特异性结合的非糖肽。随后，使用50 μL洗脱缓冲液1洗脱结合的N-糖肽，重复2次，再使用50 μL洗脱缓冲液2进行洗脱，重复2次，最后使用洗脱缓冲液3对N-糖肽进行洗脱，重复2次，收集N-糖肽，冻干，并保存在-80 ℃下，使用0.1% FA水溶液重溶后进行LC-MS/MS分析。

### 1.6 LC-MS/MS分析

#### 1.6.1 Lumos

样品采用Orbitrap Fusion Lumos质谱仪进行LC-MS/MS分析。C_18_反相毛细管柱（150 mm×150 μm，1.9 μm）；流动相A为0.1% FA水溶液，B相为含有80% ACN和0.1% FA的水溶液；梯度洗脱程序：0~8.0 min，5%B~10%B；8.0~58.0 min，10%B~24%B；58.0~70.0 min，24%B~32%B；70.0~71.0 min，32%B~95%B；71.0~78.0 min，95%B；流速为300 nL/min。

在正离子模式下采集数据。一级质谱采集质量范围为*m/z* 600~2 000，分辨率为120 000。二级质谱分辨率为50 000，隔离窗口（*m/z*）为1.6，高能碰撞解离（HCD），母离子通过30%、35%和40%的阶梯式碰撞能量进行碎裂。

#### 1.6.2 Tims TOF Pro 2

样品使用Ultimate3000液相色谱系统串联Tims TOF Pro 2质谱仪进行分析。采用C_18_反相分析柱（250 mm× 75 μm，1.9 μm）。流动相：A相为1% FA水溶液，B相为含有80% ACN和1% FA的水溶液；流速为300 nL/min。梯度洗脱程序：0~46 min，6%B~30%B；46~58 min，30%B~40%B；58~62 min，40%B~99%B。

质谱采用数据依赖型采集（DDA）模式，在正离子模式下进行，采集*m/z*为100~1 700的离子，离子传输毛细管电压为1 600 V。在平行累积连续碎裂（PASEF）模式下采集MS/MS图谱，设置如下：7次MS/MS扫描，前体离子强度阈值2 500，目标值20 000，动态排除时间24 s，碰撞能量随离子迁移率线性增加，从1/*K*_0_=0.6 Vs/cm^2^的35 eV增加到1/*K*_0_=1.6 Vs/cm^2^的65 eV，离子的累积及释放时间为100 ms；辅助气体流速：3 L/min；电离温度：180 ℃。

### 1.7 N-糖肽数据分析

#### 1.7.1 MSFragger-glyco的N-糖肽搜库方法

糖肽原始数据文件通过Fragpipe平台（version21.1）使用公开软件MSFragger-Glyco^［28］^、UniProt Human FASTA数据库（2024.06.24）及包含 252 个聚糖的 N-糖数据库进行分析。选择胰蛋白酶消化，允许两次漏切。蛋白质N端乙酰化和蛋氨酸氧化（M）被设定为可变修饰，半胱氨酸脲甲基化（C）被设定为固定修饰。在Philosopher分析流程中，肽谱匹配（PSMs）、肽段和蛋白质水平以及PTM-Shepherd 中的聚糖过滤的错误发现率（FDR）均控制在1%以下。此外，使用MSFragger（version21.1）进行N-糖肽鉴定时，设定更为严格的聚糖*p*值分界线（<0.01）。

#### 1.7.2 Maxquant的非N-糖肽搜库方法

使用Maxquant （2.2.0.0版本），根据UniProt Human FASTA数据库（2024.06.24）对糖肽原始数据进行搜索^［29］^。主要参数设定如下：（1）胰蛋白酶消化，最多含有2个漏切位点；（2）固定修饰选择半胱氨酸脲甲基化（C）；（3）可变修饰，乙酰化（蛋白质N端），蛋氨酸氧化（M）；（4）母离子质量容限和二级碎片离子质量容限分别为4.5×10^-6^和2×10^-5^；（5）肽段和蛋白质水平的假阳性率均为1%。

#### 1.7.3 N-糖蛋白的GO与KEGG Pathway分析

通过DAVID数据库对所有N-糖蛋白进行基因本体论（Gene Ontology，GO）分析和京都基因与基因组百科全书（Kyoto Encyclopedia of Genes and Genomes， KEGG） pathway分析。

#### 1.7.4 不同性别N-糖肽的差异分析

为了筛选血浆中与性别相关的N-糖肽，对N-糖肽进行统计学分析。首先对每个N-糖肽的丰度值进行转换后进行统计学检验，再以*p*<0.05和倍数变化>1.5的标准筛选出存在性别特异性的差异N-糖肽，随后对筛选出的N-糖肽进行聚类分析，定量分析不同性别组N-糖肽表达水平的差异。

## 2 结果与讨论

### 2.1 HILIC填料的考察

HILIC是N-糖肽富集的关键技术，其分离效果高度依赖于HILIC填料。理想的HILIC填料应兼具高亲水性、良好的化学稳定性及特异性结合能力，以克服血浆样本中高丰度非糖肽的干扰。亲水作用色谱填料的类型对糖肽富集效果具有显著影响。常用的填料包括中性亲水填料、两性离子型填料、阳离子型（带正电）填料、阴离子型（带负电）填料及混合模式填料，针对微量血浆样本的特殊需求，需择优选择HILIC填料，以实现高灵敏度的N-糖肽富集。我们从目前常用的HILIC填料中选择了磺酸甜菜碱型（两性离子型填料）及酰胺型（中性亲水填料）两种填料进行考察，结果如图2所示。同样1 mg的HILIC填料用量，磺酸甜菜碱型-孔径12 nm得到的N-糖肽鉴定量为1 253，磺酸甜菜碱型-孔径20 nm的N-糖肽鉴定量为1 189，而酰胺型的N-糖肽鉴定量为688，N-糖肽富集选择性分别为74.4%、73.5%和79.3%，三者的N-糖肽富集选择性相差不大。结果表明，磺酸甜菜碱型的N-糖肽富集效果优于酰胺型，两种磺酸甜菜碱型富集效果相当，无显著差别。

**图2:**
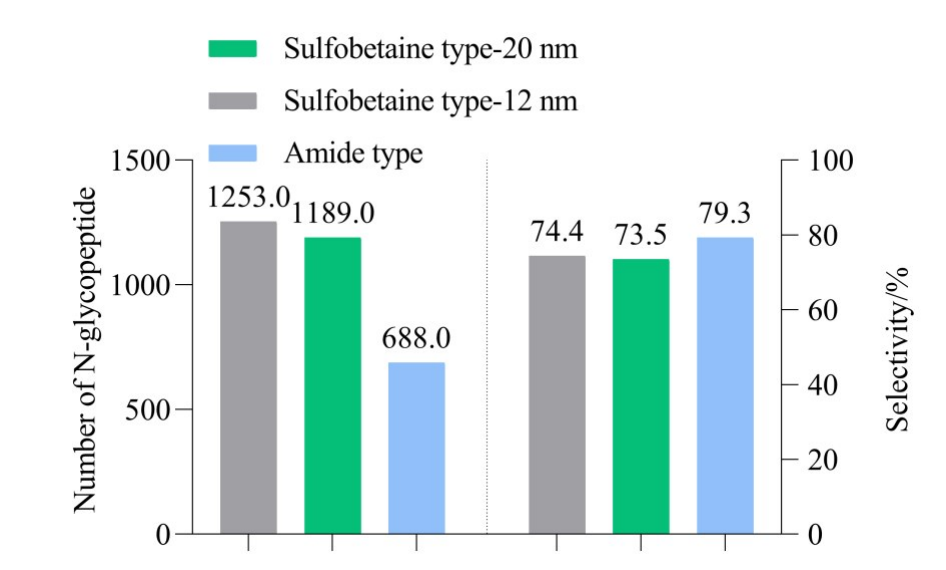
不同种类HILIC填料的N-糖肽富集鉴定水平及富集选择性

### 2.2 HILIC富集所用血浆肽段起始量的考察

在HILIC富集N-糖肽的过程中，起始肽段量对N-糖肽的鉴定效率具有显著影响。HILIC富集的微量起始肽段量考察对于稀缺样本的N-糖蛋白质组学研究具有重要意义。如图3所示，完整N-糖肽的鉴定量随着血浆起始肽段用量的增加而递增，并且N-糖肽富集的选择性也随之提高。与10 μg起始肽段量相比，使用20 μg血浆肽段（相当于0.5 μL血浆），N-糖肽鉴定量增长1.7倍，鉴定规模超过1 300种。而进一步增加起始肽段量至40 μg，N-糖肽鉴定量仅增长1.26倍。而且对于20 μg和40 μg肽段，其对应N-糖肽富集选择性都为77%左右。因此，初步选择20 μg作为后续HILIC富集的血浆肽段起始量。

**图3:**
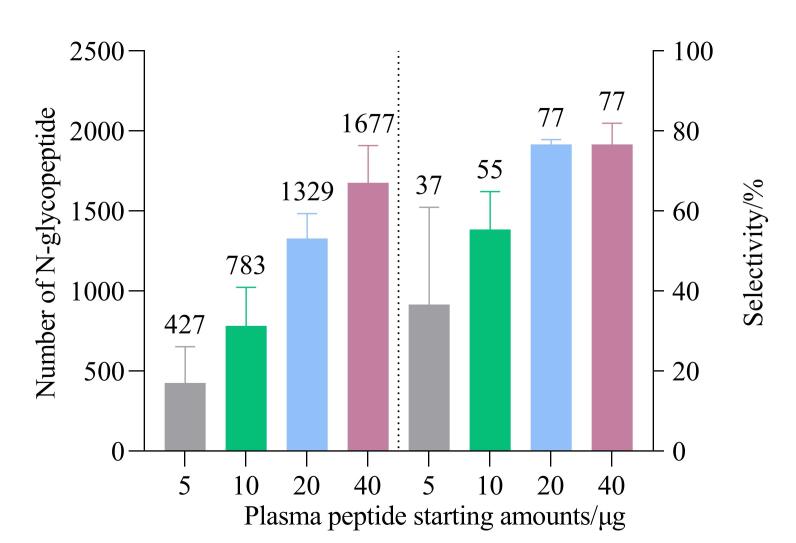
不同血浆肽段起始量进行HILIC富集时N-糖肽的鉴定水平和富集选择性

### 2.3 N-糖肽富集的洗脱梯度考察

糖肽富集是糖蛋白质组学研究中的关键步骤，HILIC富集N-糖肽的效率在很大程度上依赖于使用的溶剂。考虑到N-糖肽是N-糖链与肽段两部分组成，亲水性主要依赖于N-糖链，疏水性依赖于肽段骨架部分，为了进一步提升N-糖肽的富集效果，我们对N-糖肽洗脱溶液中乙腈的体积分数和梯度条件进行了考察。课题组常用N-糖肽洗脱梯度：使用50 μL洗脱缓冲液2进行洗脱，重复洗脱步骤3次^［21］^。通过增加洗脱缓冲液中ACN的含量，使用优化梯度（具体见1.5节）洗脱程序对N-糖肽进行洗脱，结果如图4所示，在3次重复实验中，优化后的洗脱条件下平均富集鉴定到1 663种N-糖肽，而原洗脱条件的平均鉴定量为1 329种，N-糖肽鉴定量增加1.25倍。虽然优化后的富集条件由于乙腈比例提高使少量非糖肽也被洗脱，导致N-糖肽富集选择性轻微下降，但相差不大。因此选择优化后的洗脱条件进行后续方法考察。

**图4:**
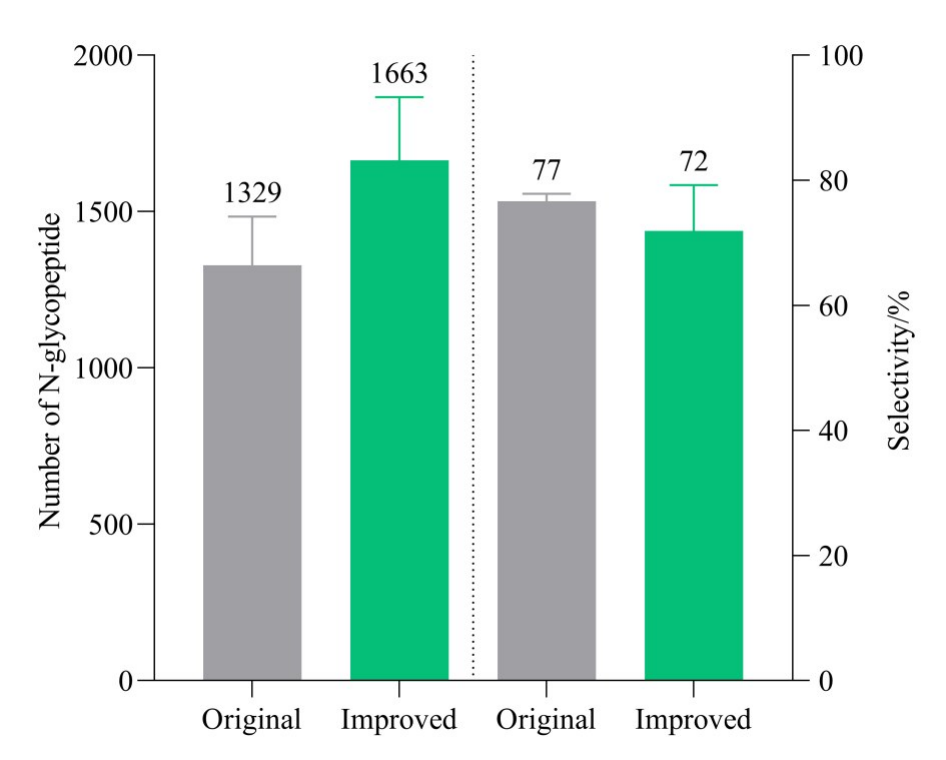
不同洗脱条件所得N-糖肽的鉴定水平和富集选择性（*n*=3）

### 2.4 Tims TOF Pro 2与Lumos质谱的N-糖肽鉴定效果对比

液相色谱-质谱技术是糖蛋白组研究领域的金标准，但传统质谱仪扫描速度和灵敏度有限，在分析复杂生物样品时仍面临糖蛋白组深度覆盖的挑战。基于平行累积序列碎片技术的 Tims TOF Pro 2质谱仪具有高稳定性和高灵敏度，为四维多组学的应用和发展拓宽了道路^［30，31］^。随着捕获离子淌度技术的引入，增加了新的分离维度，提高了区分异构体的信噪比，并开启了全新的并行累积序列碎片技术，实现了极高的二次扫描速度，并且只需少量样品即可实现N-糖蛋白组鉴定的深度覆盖。如图5所示，经过3次重复实验，使用MSFragger进行搜库，Lumos质谱仪进行检测，平均每次鉴定得到1 289条N-糖肽，116个N-糖蛋白；使用Tims TOF Pro 2质谱仪进行检测，平均每次鉴定到1 804条N-糖肽与149个N-糖蛋白，N-糖肽鉴定量增加1.4倍，N-糖蛋白增加1.3倍。对比Lumos与Tims TOF Pro 2的N-糖肽鉴定结果（图6），Tims TOF Pro 2鉴定得到1 126条独有N-糖肽，71个独有N-糖蛋白，占总N-糖肽的38.01%，总N-糖蛋白的33.65%；Lumos鉴定得到592条独有N-糖肽，22个独有N-糖蛋白，占总N-糖肽的19.99%，总N-糖蛋白的10.43%，以上结果表明Tims TOF Pro 2鉴定血浆N-糖蛋白的效果优于Lumos。

**图5:**
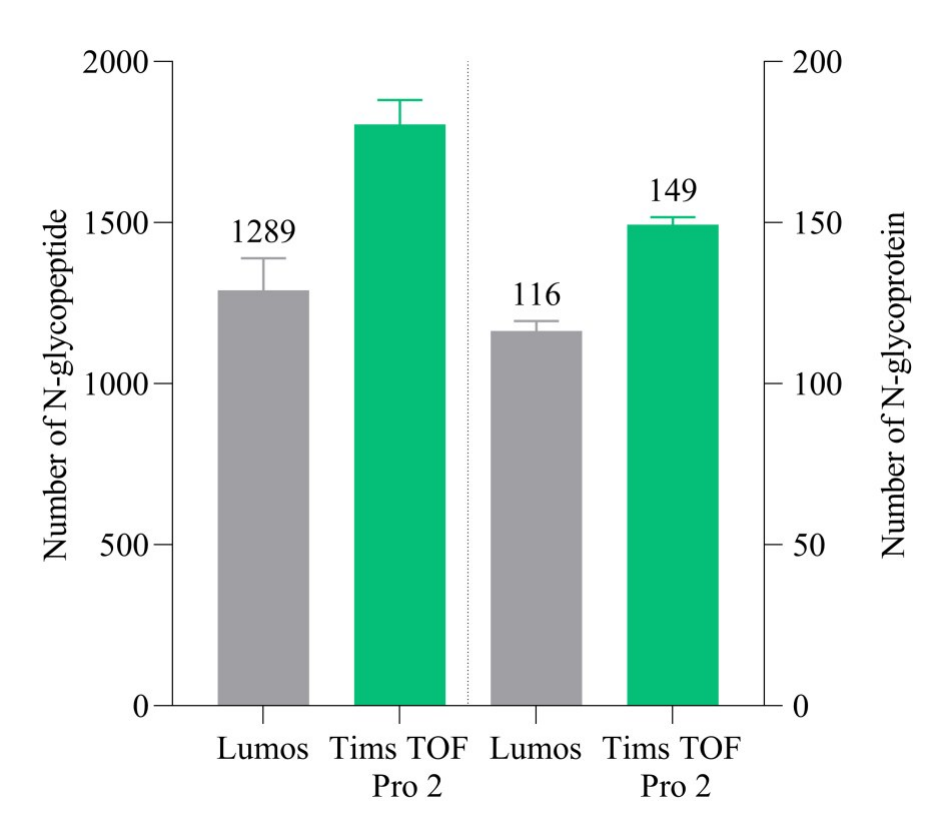
Lumos与Tims TOF Pro 2 质谱鉴定到的N-糖肽与N-糖蛋白数量（*n*=3）

**图6:**
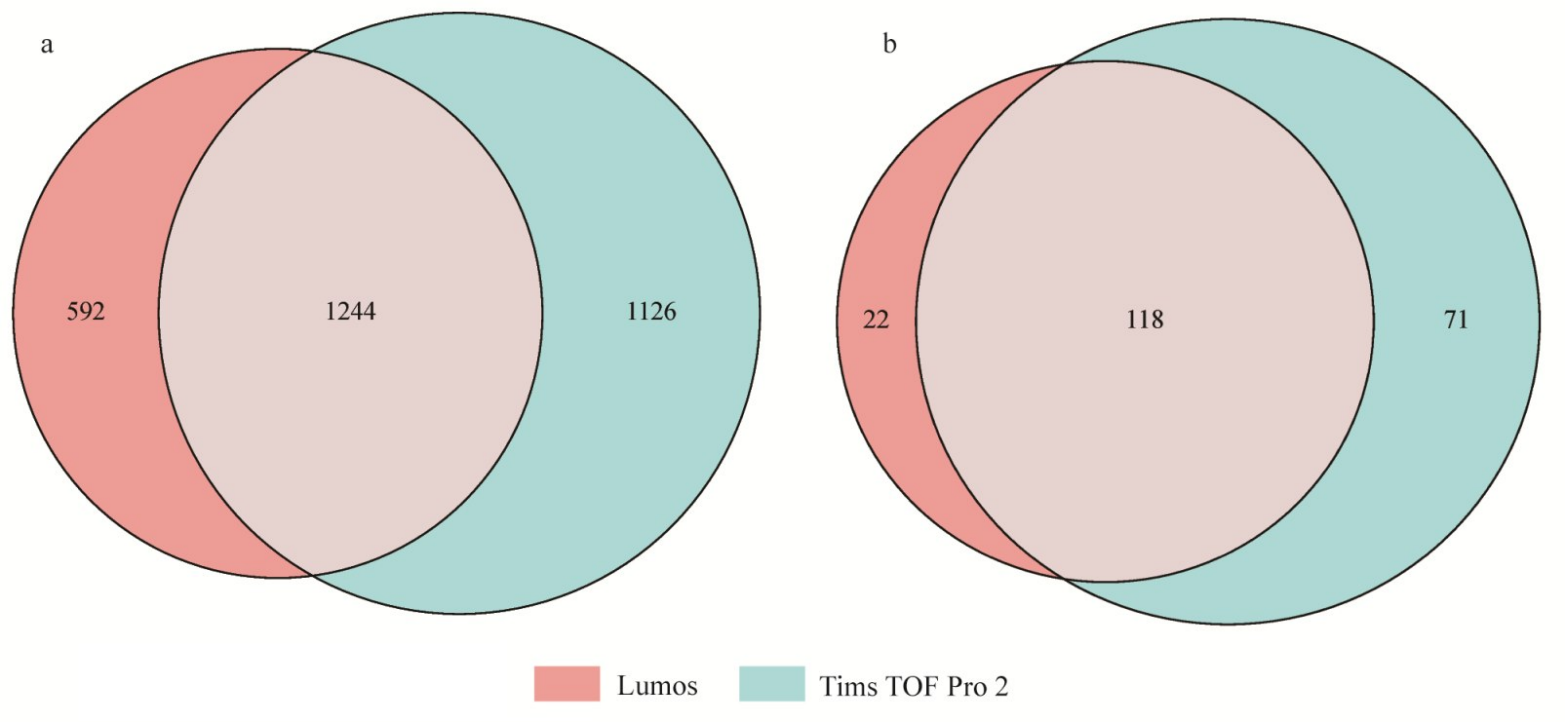
Lumos与Tims TOF Pro 2质谱鉴定的（a）N-糖肽和（b）N-糖蛋白的韦恩图

本工作通过Lumos与Tims TOF Pro 2两种仪器一共鉴定得到2 962条N-糖肽与211个N-糖蛋白，使用DAVID数据库对鉴定到的N-糖蛋白进一步进行GO富集分析，以确定其定位、分子功能和生物学过程。如图7所示，我们发现这些N-糖蛋白参与各种生物学过程，如补体激活、先天性免疫反应和凋亡细胞的清除等过程；大部分定位于攻膜复合物、循环免疫球蛋白复合物和IgG免疫球蛋白复合物；主要的分子功能包括整合素结合、胶原蛋白结合及免疫球蛋白受体结合，这些结果表明N-糖蛋白或N-糖基化在人体循环及免疫系统中起着至关重要的作用。此外，KEGG通路分析结果（图8）显示，细胞外基质-受体相互作用、血小板活化与胆固醇代谢通路、造血细胞谱系通路与磷脂酰肌醇3激酶-蛋白激酶B信号通路显著富集，提示N-糖蛋白可能参与细胞的黏附、迁移、分化和增殖活动，介导血小板聚集与止血栓形成以及调节体内胆固醇代谢的平衡，对理解血液系统发育、免疫功能和血液疾病至关重要，并且参与多种生理过程，能调控细胞存活、转移和新陈代谢，在血管生成和炎症因子募集中发挥作用。

**图7:**
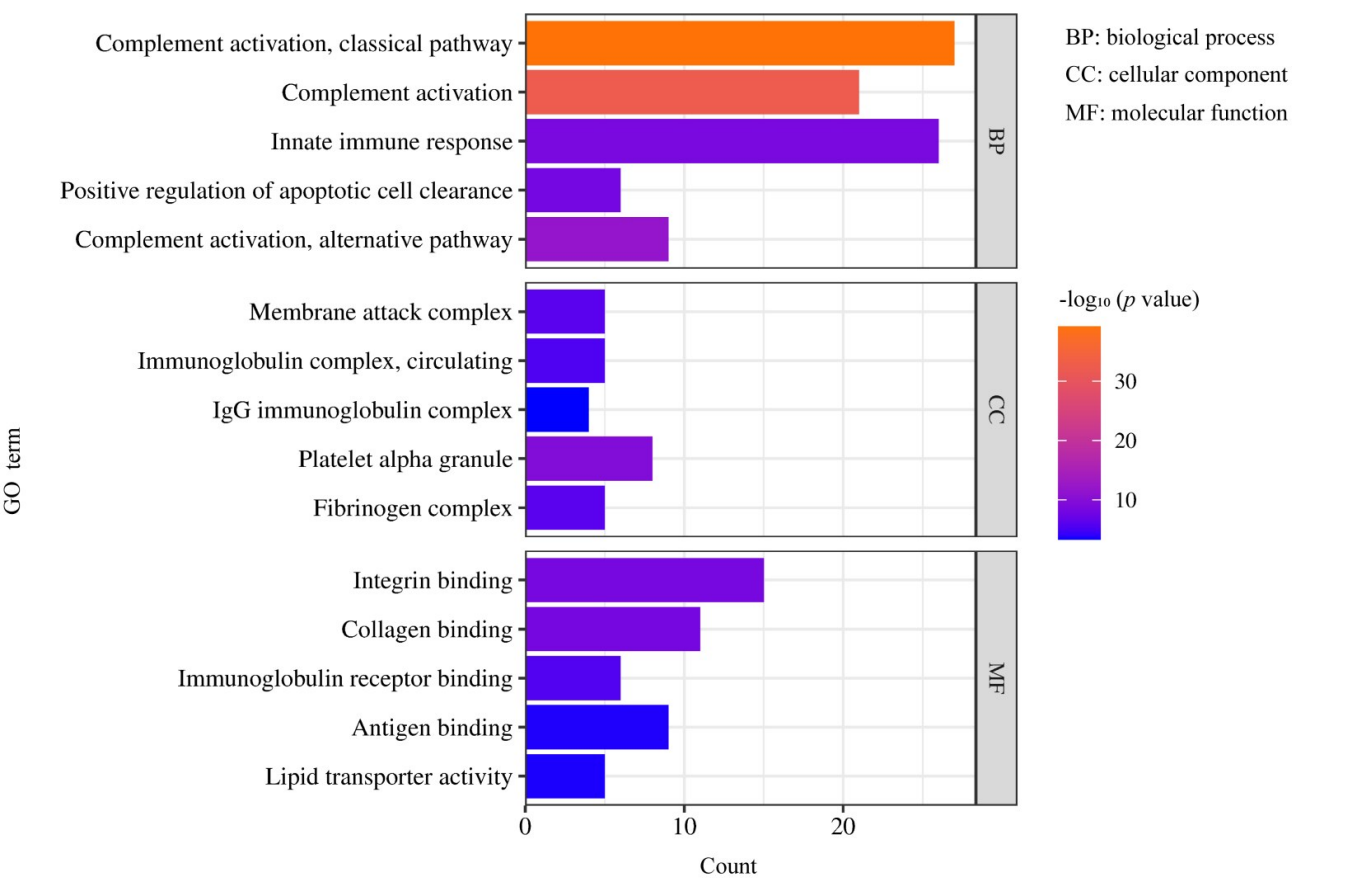
本工作鉴定N-糖蛋白的GO分析

**图8:**
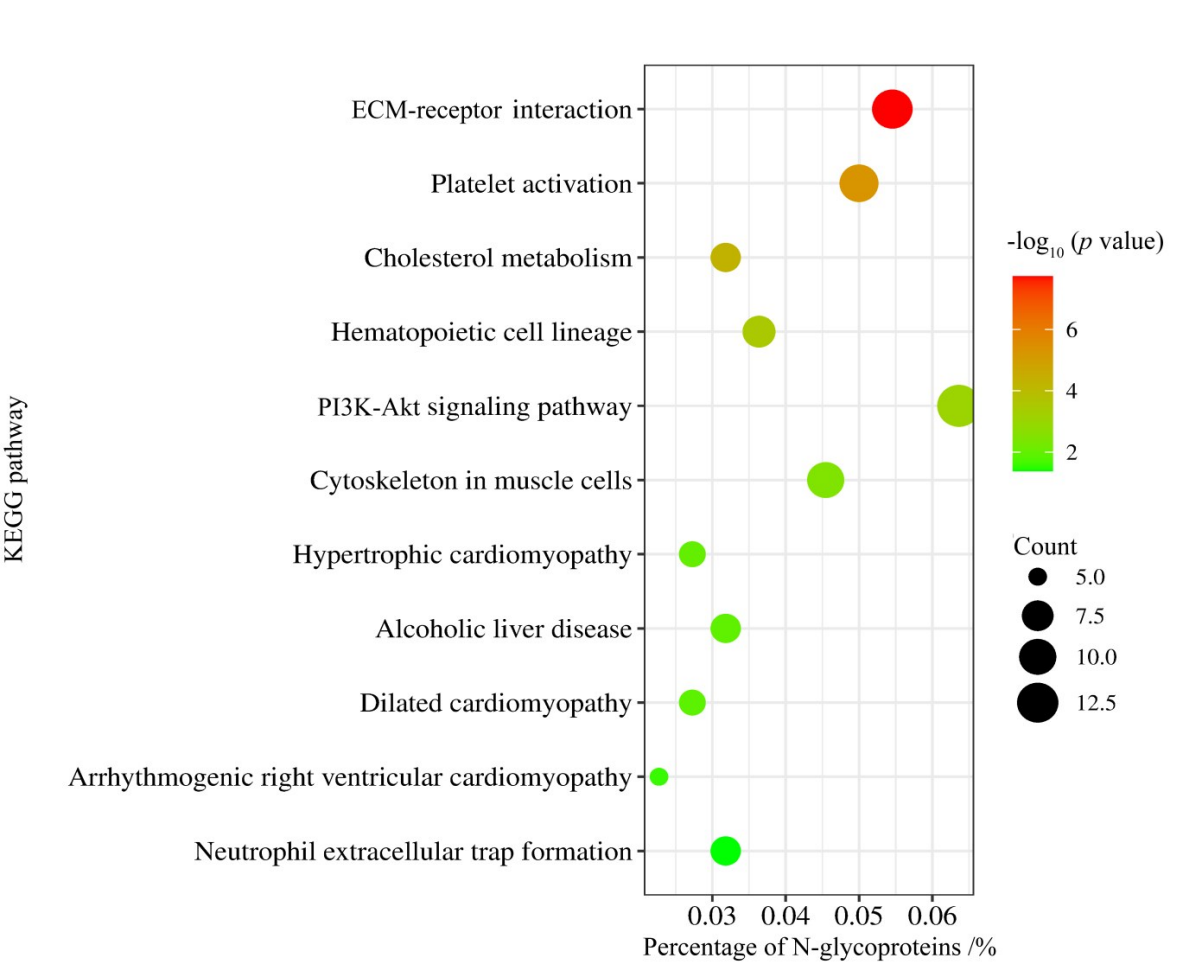
本工作鉴定N-糖蛋白的KEGG pathway分析

### 2.5 不同性别血浆样本差异N-糖肽分析

通过分析6例健康志愿者血浆N-糖肽（3例健康女性，3例健康男性）的表达情况，我们共鉴定到20个差异表达N-糖肽。如图9a所示，横坐标为女性与男性N-糖肽定量值倍数比的对数值，纵坐标表示*p*值的负对数。其中绿色代表相比于女性，男性显示下调的N-糖肽，红色代表相比于女性，男性显示上调的N-糖肽。男性中有16个N-糖肽相比于女性明显上调，有4个N-糖肽相比于女性显著下调。接下来对筛选到的差异N-糖肽的表达水平进行聚类分析。如图9b显示，两者N-糖肽的表达量显示出明显的性别差异，说明性别可能是正常个体血浆中N-糖肽存在差异的一项重要因素，在临床诊断及生物标志物的筛选中应予以考虑。

**图9:**
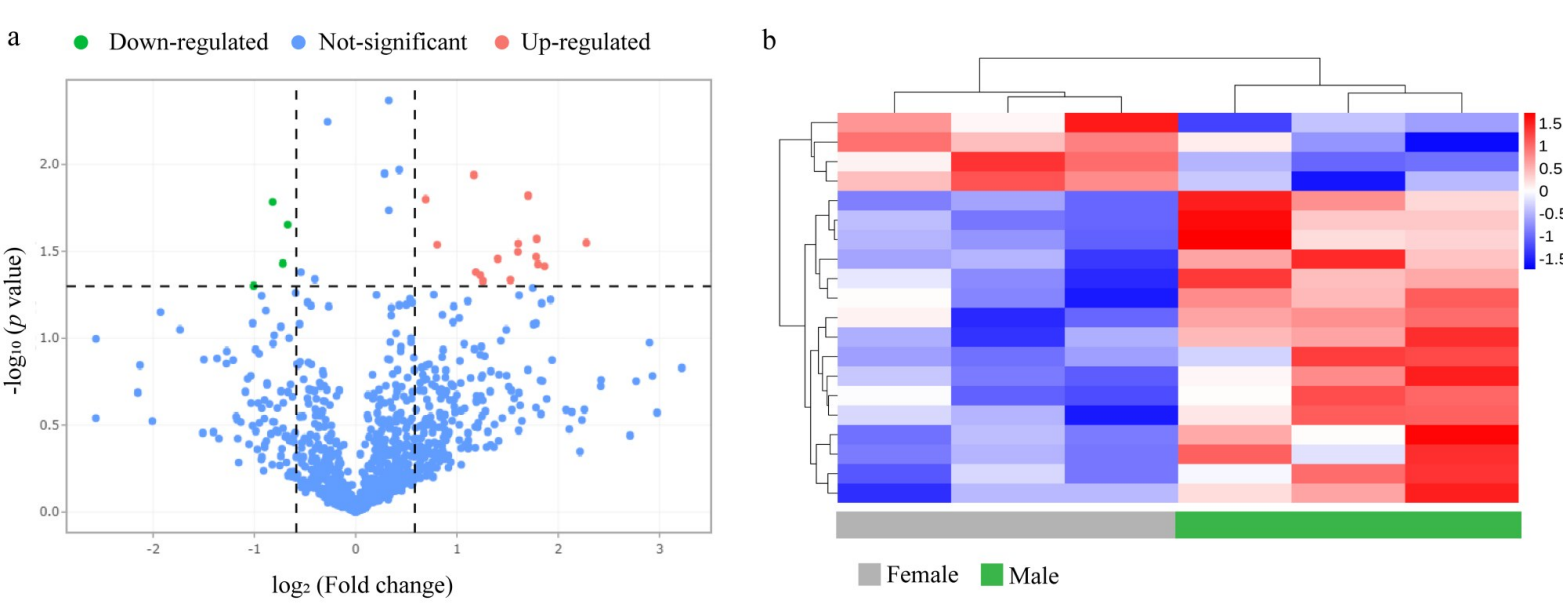
（a）依赖于性别差异表达的N-糖肽的火山图与（b）差异表达水平热图

## 3 结论

本研究通过系统优化N-糖肽富集与检测技术路线，建立了适用于微量血浆的高效N-糖蛋白质组分析平台，采用磺酸甜菜碱型HILIC填料，实现高效N-糖肽富集，并且在富集选择性基本不变的条件下，优化了N-糖肽洗脱梯度，使N-糖肽鉴定量增加至1.25倍。同时整合Tims TOF Pro 2的离子淌度分离与Orbitrap Lumos的高分辨率检测，形成互补分析策略，在仅使用20 μg血浆肽段（等效0.5 μL全血浆）的条件下可稳定鉴定出211种N-糖蛋白和2 962种N-糖肽。为≤1 μL血浆样本量发现糖基化生物标志物提供依据，提高了生物标志物的检测灵敏度，对推动精准医学研究和临床诊断具有重要意义。
